# Socioeconomic status and self-rated health in China: Findings from a cross-sectional study: Erratum

**DOI:** 10.1097/MD.0000000000016901

**Published:** 2019-08-16

In the article, “Socioeconomic status and self-rated health in China: Findings from a cross-sectional study”,^[1]^ which appears in Volume 98, Issue 12 of *Medicine*, Tong Yu's degree appeared incorrectly and should be Master of Law (ML), not PhD.
